# Symptomatic treatment of the common cold with a fixed-dose combination of paracetamol, chlorphenamine and phenylephrine: a randomized, placebo-controlled trial

**DOI:** 10.1186/1471-2334-13-556

**Published:** 2013-11-22

**Authors:** Paulo Dornelles Picon, Marisa Boff Costa, Rafael da Veiga Picon, Lucia Costa Cabral Fendt, Maurício Leichter Suksteris, Indara Carmanim Saccilotto, Alicia Dorneles Dornelles, Luis Felipe Carissimi Schmidt

**Affiliations:** 1 Department of Internal Medicine, Universidade Federal do Rio Grande do Sul (UFRGS), Porto Alegre, RS, Brazil; 2 Center for Clinical Research, Hospital de Clínicas de Porto Alegre (HCPA), Universidade Federal do Rio Grande do Sul (UFRGS), Porto Alegre, RS, Brazil

**Keywords:** Efficacy, Safety, Symptoms, Common cold

## Abstract

**Background:**

The common cold and other viral airway infections are highly prevalent in the population, and their treatment often requires the use of medications for symptomatic relief. Paracetamol is as an analgesic and antipyretic; chlorphenamine is an antihistamine; and phenylephrine, a vasoconstrictor and decongestant. This randomized, double-blind, placebo-controlled trial sought to evaluate the efficacy and safety of a fixed-dose combination of paracetamol, chlorphenamine and phenylephrine in the symptomatic treatment of the common cold and flu-like syndrome in adults.

**Methods:**

This study enrolled 146 individuals aged 18 to 60 years who had moderate to severe flu-like syndrome or common cold. After clinical examination and laboratory tests, individuals were randomly assigned to receive the fixed-dose combination (73) or placebo (73), five capsules per day for 48 to 72 hours. The primary efficacy endpoint was the sum of the scores of 10 symptoms on a four-point Likert-type scale. To evaluate treatment safety, the occurrence of adverse events was also measured.

**Results:**

Mean age was 33.5 (±9.5) years in the placebo group and 33.8 (±11.5) in the treatment group. There were 55 women and 18 men in the placebo group, and 46 women and 27 men in the treatment group. Comparison of overall symptom scores in the two groups revealed a significantly greater reduction in the treatment group than in the placebo group (p = 0.015). Analysis at the first 13 dose intervals (± 66 h of treatment) showed a greater reduction of symptom scores in the treatment group than in the placebo group (p < 0.05). The number and distribution of adverse events were similar in both groups.

**Conclusion:**

A fixed-dose combination of paracetamol, chlorphenamine and phenylephrine was safe and more effective than placebo in the symptomatic treatment of the common cold or flu-like syndrome in adults.

**Trial registration:**

NCT01389518

## Background

Acute respiratory infections are highly prevalent in the population, with the common cold, flu-like syndromes, tracheobronchitis, sinusitis, laryngitis and pneumonias being particularly important. When the aetiology is presumably bacterial, treatment is based on the use of antibiotics and medication for symptomatic relief. However, the most common clinical entities, the common cold and the flu-like syndrome, have a viral aetiology, for which symptomatic treatment remains, in most cases, the standard recommendation.

The common cold is the most frequently encountered disease in medical practice [1]. It affects most adults, on average two to four times a year, and accounts for up to 40% of work absences among the economically active population in the United States [2].

This syndrome affects the upper airways, sometimes in association with low-grade fever and systemic symptoms, and usually presents with at least two of the following symptoms: cough, dysphonia, throat discomfort, sore throat, nasal congestion, rhinorrhoea, sneezing, headaches, myalgia and fever [3]. Symptoms usually peak at 2 to 3 days and have a mean duration of 7 to 10 days [4]. This definition has been prospectively validated in other studies and is that most frequently used in clinical studies of patients with the common cold [5]. Although most cases are caused by the rhinovirus, other agents may be involved, such as the respiratory syncytial virus, adenovirus, coronavirus, and influenza and parainfluenza viruses [6].

Flu-like syndrome is characterized by sudden onset of fever, headache, cough, sore throat, myalgia, nasal congestion, weakness and loss of appetite [4]. Complications, such as pneumonia, otitis and sinusitis, may occur [7].

Some of the medications studied for the treatment of the common cold are antihistamines, anticholinergics, alpha-adrenergic agonists, membrane stabilizers, nonsteroidal anti-inflammatory drugs, vitamin C, glucocorticoids, zinc, herbal medications and alpha-interferon. Clinical trials of high-dose vitamin C have not found any benefits in the treatment of the common cold [8]. Recent studies of *Echinacea purpurea*, a herbal medicine, did not find any clinical benefits for patients with the common cold [9-12]. The results of meta-analyses about the efficacy of zinc are contradictory, and there is weak evidence of its benefit in well-designed studies [13-15].

The symptomatic treatment of the common cold has been evaluated in Cochrane meta-analyses. The first included 32 studies with a total of 8930 patients and investigated the administration of antihistamines in the common cold. Results showed that monotherapy did not improve symptoms in either children or adults [16]. The combined use of antihistamines and decongestants may alleviate symptoms in adults, but results are heterogeneous [15,17]. Another meta-analysis investigated the use of nasal decongestants in the common cold in 286 adults, and found no benefit for the relief of nasal congestion [15,18]. Another recent meta-analysis [19] suggests that a triple combination of antihistamine, decongestant and analgesic provided some general benefit in adults and older children.

Most cases of influenza require the use of drugs for symptomatic relief [1,20]. In clinical practice, treatment directed to the aetiological agent is not routine, but became considerably more frequent after the H1N1 pandemic in 2009 [21].

Therefore, patients continue to use medications that produce symptomatic relief because of the epidemiological relevance and the intensity of symptoms of flu-like syndromes and the common cold.

This study evaluated the efficacy and safety of a fixed-dose combination of paracetamol, chlorphenamine and phenylephrine in the symptomatic treatment of the common cold and flu-like syndromes by analysing symptom score reductions during and after treatment, duration of symptoms, return to usual activities and adverse events in both groups.

No other randomized clinical trial (RCT) evaluated the safety and efficacy of this specific fixed-dose combination for the common cold in adults. This was the first RCT conducted in Brazil designed to also assess adverse cardiovascular effects of phenylephrine.

## Methods

This phase III, prospective, randomized, double-blind, placebo-controlled clinical trial enrolled volunteers recruited by means of posters displayed in Hospital de Clínicas de Porto Alegre (HCPA) and in primary care centres in the city of Porto Alegre, Brazil, and of ads placed in local newspapers.

### Inclusion criteria

This study included volunteers of both genders, aged 18 to 60 years, with a duration of symptoms no longer than 72 hours. The common cold was defined by the presence of at least two of the following 10 symptoms: sneezing, rhinorrhoea, nasal congestion, headache, myalgia, throat discomfort, sore throat, dysphonia, cough and fever. Symptoms should be moderate to severe on a four-point, Likert-type symptom severity scale (0 = no symptoms; 1 = mild; 2 = moderate; 3 = severe). Flu-like syndrome should include fever of at least 38.1°C and moderate to severe headache or moderate to severe myalgia or arthralgia in the Likert-type symptom severity scale described above.

### Exclusion criteria

Volunteers were excluded from the study if they met any of the following criteria: pregnancy or breastfeeding; known hypersensitivity to any component of the study formulation; use of alcohol or illicit drugs; use of monoamine oxidase (MAO) inhibitors or barbiturates; perennial or seasonal allergic rhinitis confirmed at screening; any current acute disease or uncontrolled exacerbation of chronic disease; clinical evidence of immunosuppression; vaccination against influenza up to 1 week before inclusion; need for antiviral therapy to treat influenza A or B infection; need for antibacterial therapy to treat acute respiratory infection; use of medication to treat conditions acquired before inclusion for a time shorter than two time intervals of administration of these drugs; and participation in another clinical trial less than 1 year before.

### Sample size calculation

For a statistical power of 80%, a random error of 5% and a response rate of 30% in the treatment group in comparison with the placebo group, and a 50% reduction in symptom scores after the second day of follow-up in the placebo group, the sample size was calculated as 132 patients (66 in each group) [22]. To account for a possible loss to follow-up of 10% of the volunteers, 146 patients were included in the study (73 in each group).

### Statistical analysis

All variables were collected and entered into the database before randomization codes were opened. The database was created using the Epi-INFO 3.5.1 software. SPSS 14.0 was used for statistical analyses.

All variables of interest were expressed as absolute and relative frequencies. The Student *t*-test for independent samples was used for the comparison of means for all continuous, symmetrically distributed variables. Repeated-measures analysis of variance (ANOVA) was used for the analysis of variance of overall score means in 11 measurements.

All randomized patients were included in the analysis of efficacy (intention to treat). For all analyses, the level of significance was set at p = 0.05.

### Randomization and blinding

Randomization for this clinical trial was performed using the Random Allocation Software and simple randomization into two groups by means of a table of random numbers, which generated a simple randomization spreadsheet.

The list of random numbers was placed in a sealed envelope kept by the statistician responsible for analyses. None of the authors had access to the randomization table. The active drug and placebo capsules had identical organoleptic characteristics. A standard label, designed for use with both groups, contained the following information: name of the study, study registration number with of HCPA Research Ethics Committee, bottle number/randomization number, and instructions for use and storage.

### Intervention

Study drug: One capsule of a fixed-dose combination of 400 mg paracetamol, 4.0 mg chlorphenamine and 4.0 mg phenylephrine was administered according to manufacturer instructions [23] every 4 hours during waking hours between 7 a.m. and 11 p.m. (five daily doses) for 2 to 3 consecutive days. Patients were instructed to use the drug for no fewer than 2 and no more than 3 days as this reflects best clinical practice, in which users decide the maximum duration of use according to the intensity of their symptoms.

Placebo: One capsule was administered every 4 hours during waking hours between 7 a.m. and 11 p.m. (five daily doses) for 2 to 3 consecutive days.

Each participant included in the study received a bottle containing 15 capsules of the study drug or placebo, depending on group allocation, and one bottle containing 12 tablets of rescue medication (500 mg paracetamol).

### Procedures

The study lasted 10 days and included three clinical visits (V1, V2 and V3) and two rounds of laboratory tests (baseline and V2).

In addition to medical history and physical examination, the participants underwent laboratory tests and electrocardiograms (ECG). Immediately afterwards, they were randomly allocated to one of the two groups and received the study drug or placebo, a diary to keep note of symptoms and adverse events, and the rescue medication.

At the end of treatment, which lasted 2 to 3 days, the patients returned for re-evaluation. The second visit included medical history, clinical and physical examination, ECG and laboratory tests, as well as an analysis of the following aspects: symptoms, using pre-defined scales; adverse events; duration of symptoms; time to return to usual activities; and drug intake, by counting the capsules and tablets used.

The third and last follow-up visit took place 7 days after the end of the treatment, when the following parameters were evaluated: persistent symptoms, according to predefined scales; duration of symptoms; time to return to usual activities; adverse events; and use of other medications after stopping use of the study drug.

Group allocation was disclosed only after data had been collected and entered into the study database, by double and independent entry followed by a comparison of the two datasets. The envelope containing the randomization table was opened in the presence of representatives of the study sponsor and the principal investigator.

Data were collected for this clinical trial from June 2, 2009 to July 7, 2009. The clinical visits were conducted at the HCPA Clinical Drug Research Center (NUCLIMED). The database was checked and analysed and the final report was written from August 8 to October 31, 2009.

### Efficacy analysis

The primary endpoint was the sum of the scores of 10 common cold or flu-like syndrome symptoms evaluated on a four-point Likert-type scale for intensity (severity): 0 = no symptoms; 1 = mild; 2 = moderate; and 3 = severe. The maximum and minimum scores for each measurement were 40 and zero respectively. This scale was used by the physician during visits and also by the patients for daily self-evaluation using the study diary. Participants rated their symptoms at home before each administration of the study drug or placebo throughout the treatment period, so that a total of 10 to 15 measurements were obtained.

Secondary endpoints were overall symptom duration, time to return to usual activities, and use of rescue medication.

### Safety analysis

Drug safety was evaluated by the occurrence of adverse events detected in clinical history, physical examination findings, or abnormal lab results during treatment or up to 7 days after the last dose of the medication.

All serious and non-serious adverse events were fully documented using clinical charts, original documents, and specific forms. In addition, all adverse events occurring within 7 days of the treatment were investigated, recorded and compared between groups. Adverse events were followed until their resolution or until follow-up was classified (in writing) as complete by the investigators.

### Bioethical issues

This study was approved by the Ethics Committee of the Graduate Studies and Research Group of Hospital de Clínicas de Porto Alegre (HCPA). All participants were volunteers, and all procedures for this study were started only after participants had read and signed an informed consent form approved by the HCPA Research Ethics Committee.

## Results

**Figure 1:**
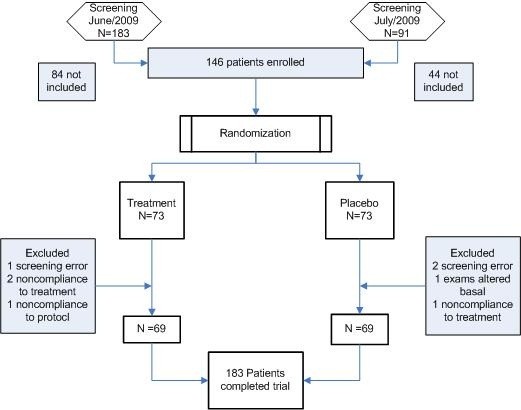
Study flowchart.

This study included 146 patients randomly distributed into two groups (placebo and treatment) of 73 patients each (Figure 1).

Table 1 shows the baseline characteristics of the study sample. The groups were homogeneous except for weight, which was greater in the treatment group. Mean (±SD) time between V1 and V2 was 72.9 (±10.5) hours, with no significant between-group differences (p = 0.919).

**Table 1:** Baseline characteristics of the study population according to group (placebo or treatment)

	**Placebo group**	**Treatment group**
Age (years)	33.5 (±9.46)	33.82 (±11.48)
Sex		
Female	55	47
Male	18	26
Height (m)	1.64 (±0.08)	1.67 (±0.11)
BMI (kg/m^2^)	25.28 (±4.29)	26.95 (±6.03)
Mean symptom score	14.23 (±4.09)	14.89 (±3.78)
Weight (kg)	68.21 (±13.18)	75.27 (±18.53)
Time from symptom onset to start of treatment (h)	42.29 (±17.54)	46.57 (±15.65)

### Efficacy analysis

Mean symptom scores were calculated for both groups at V1 and V2 (before and after treatment), without any data loss, as 100% of the patients attended both visits.

**Figure 2:**
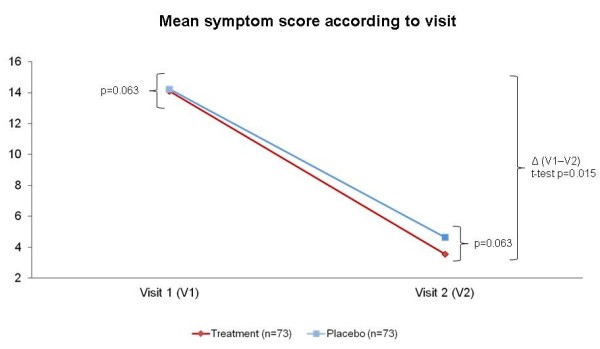
**Mean symptom score before and after treatment.** * Comparison between mean overall scores: Student *t*-test for independent samples; ** Mean score variation in the two groups (V1-V2): Student *t*-test for independent samples.

At V1, the mean (±SD) overall symptom score was 14.23 (±4.09) in the placebo group and 14.09 (±3.78) in the treatment group, which demonstrates similarity between groups at that time point. At V2, the overall symptom score was markedly reduced in both groups, from 14.23 to 4.64 in the placebo group and from 14.09 to 3.54 in the treatment group. Separate comparison of mean overall scores of symptoms as evaluated by physicians at V2 showed that scores were lower in the treatment group, with a trend toward statistical significance (p = 0.063). Comparison of the overall score reduction induced by treatment or placebo (V1-V2) in the two groups revealed that this reduction was significantly greater in the treatment group (p = 0.015) (Figure 2). The total number of days with reported symptoms was measured in 55 and 49 subjects of the placebo and treatment groups respectively. The mean (+SD) number of days with symptoms was 7.5 (+5.3) days in the placebo group and 5.9 (+3.4) days in the active treatment group (p = 0.08).

**Figure 3:**
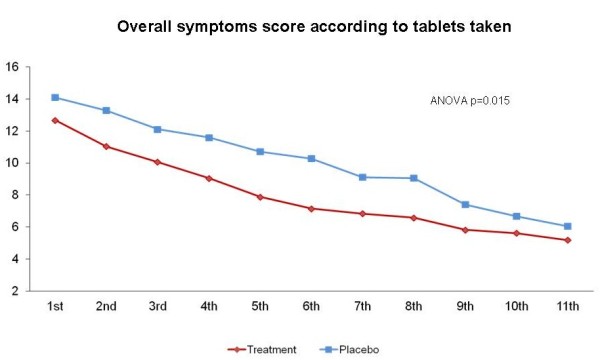
**Overall patient-reported score in each group (treatment vs. placebo) at 11-dose intervals.** * Repeated-measures analysis of variance (ANOVA).

Analysis of the symptom score curves of both groups revealed that the curves separated after the second dose. ANOVA showed that, during the first 11 dose intervals (Figure 3), which corresponded to at least 52 hours of treatment, a statistically significant reduction in symptom scores occurred in the treatment group in comparison with the placebo group (p < 0.049). ANOVA including the first 13 dose intervals, a time corresponding to 66 hours of treatment (including hours of sleep), also revealed a reduction in symptom scores in the treatment group, again with a statistically significant difference (p ≤ 0.05).

To evaluate the use of rescue medication (500 mg paracetamol), the mean number of tablets taken and the total number of patients that used the rescue medication in each group were compared. The mean number of tablets taken was lower in the treatment group than in the placebo group (0.78 [±1.88] vs. 1.3 [±1.72]; p = 0.002). A greater percentage of patients in the placebo group used rescue paracetamol (50.7%) than in the treatment group (25%), and this difference was statistically significant (chi-square test, p = 0.001).

Mean axillary temperature was not statistically different between groups, and there were no differences between axillary temperature values at V1 and V2.

### Safety analysis

The overall incidence of patient-reported adverse events was 34 events in the placebo group and 39 in the treatment group, with no statistically significant between-group differences (p = 0.508). Furthermore, analysis of the incidence of specific symptoms in each group did not reveal any statistically significant differences, and the distribution of the number of adverse events was homogeneous in the two groups. The main adverse event reported was drowsiness (n = 43), followed by nausea (n = 18). Overall, 110 patients experienced some type of adverse effect in both groups.

Mean (±SD) heart rate (HR) was 76.60 (±11.04) bpm in the treatment group and 77.36 (±12.96) bpm in the placebo group at V1. At V2, mean HR was 76.72 (±10.53) bpm in the treatment group and 77.96 (±11.00) bpm in the placebo group. Comparisons of HR variation before and after treatment did not reveal any significant between-group differences (p = 0.834).

Mean (±SD) systolic blood pressure (SBP) at V1 was 119.22 (±13.09) mmHg in the treatment group and 115.66 (±13.55) mmHg in the placebo group, and this difference was not significant (p = 0.11). At V2, mean SBP was 120.82 (±15.93) mmHg in the treatment group and 114.01 (±12.29) mmHg in the placebo group, and this difference was statistically significant (p = 0.004). Comparison of BP variation before and after treatment did not reveal any significant differences (p = 0.092).

Laboratory test results did not show any clinically relevant changes in relation to any of the study variables. β-HCG, urinalysis (density, pH, proteinuria, glycosuria, ketonuria, bilirubin, urobilinogen, haemoglobinuria, nitrite, leukocyte esterase) and FBC results were not significantly different between V1 and V2.

No serious adverse events were recorded during the study, and no patients withdrew from the study due to adverse events. The reasons for exclusion from the sample are shown in Figure 1.

## Discussion

This was the first randomized, double-blind clinical trial to demonstrate the efficacy of a fixed-dose combination of 400 mg paracetamol, 4.0 mg chlorphenamine and 4.0 mg phenylephrine in reducing flu-like symptoms by means of both objective evaluation of symptoms by the investigators and subjective reports provided by patients. The evaluation of rescue paracetamol use as a dichotomous variable showed that 50.7% of patients in the placebo group versus 25% in the treatment group used the rescue medication. This difference indicates that for every four patients treated with the study drug, one did not take rescue paracetamol (NNT = 4). This reduction of the rate of rescue medication use is an index of analgesic efficacy and demonstrates that patients with flu-like symptoms actually seek an alternative form of relief when the medication they are taking is not efficacious. Time to freedom from symptoms was lower in the treatment group than in the placebo group, although the difference did not reach statistical significance.

The comparison of baseline and final scores revealed that both groups experienced a major reduction in the total symptom score, both as assessed by the investigators and as reported by the patients themselves, a result that may be attributed to the fact that the common cold is a self-limiting disease. The rapid symptom reduction precludes demonstration of any between-group differences at the end of the treatment (48 to 72 hours). Therefore, the fact that treatment group had a statistically significant difference from the placebo group at this time point supports the efficacy of the study drug.

Evaluation of patient-reported symptom scores (study diary) by ANOVA showed a statistically significant reduction in symptom scores in the treatment group as compared with the placebo groups (p ≤ 0.05). This difference of up to 3 points in symptom scores was constant during the first 13 doses and translates into a trend towards a 1.5-day reduction in the total duration of symptoms. If confirmed in larger studies, this finding could have a major impact on the population, given the high annual incidence of flu-like syndrome and the large number of users of over-the-counter medication for symptomatic relief of its symptoms worldwide. After the 13th dose, symptom scores were very low and similar between groups, which confirms the self-limiting characteristic of the disease and made it difficult for patients to assign scores to their symptoms. According to the study protocol, patients did not have to take the medication (either active or placebo) for more than 10 doses (corresponding to 48 hours, or 2 days). This explains the reduction in the number of cases for analysis after the 13th dose.

Analysis of the HR variation attributable to treatment revealed that the active formulation did not increase HR. Furthermore, treatment did not increase systolic BP. A small between-group difference in this variable was already detectable at baseline. Although not significant, patients in the treatment group had higher baseline mean values (a 4.44 mmHg difference), which may be attributable to randomization. However, all mean values in both groups were within normal limits both at baseline and after treatment. The increase in mean systolic BP attributable to treatment with the active formulation was only 1.6 mmHg; therefore, there was no increase in sensitivity to sympathomimetic agents, and the response after treatment in this group was not more intense than in the placebo group. The absence of patient- or investigator-detected effects on cardiovascular parameters, HR, or SBP suggests that the study drug did not increase cardiovascular risk. Hence, this RCT adds valuable safety data, particularly in regard to the effects of the decongestant agent phenylephrine on blood pressure, heart rate and ECG parameters.

Some limitations of this study must be mentioned, including the difficulties of studying a disease such as the common cold, which is self-limited and combines multiple symptoms with a very rapid rate of resolution. The majority of patients were symptom-free by the 7th day after the end of treatment. Moreover, most patients had a low symptom score at randomization, probably because of the generally accepted concept of at-home recovery for flu-like illness. This limitation had a negative influence on identification of severe disease, which, combined with the rapid resolution of symptoms (very low symptom score), reduces the possibility of identifying between-group differences. For these reasons, any statistically significant differences found might be interpreted as having potential for clinical relevance.

Safety analysis did not reveal any clinically relevant change in any clinical or laboratory variables at the two time points of evaluation, whether within-group or between-group (active vs. placebo). Furthermore, there were no statistically significant between-group differences in treatment-emergent adverse effects.

## Conclusion

Administration of the study drug at the dose suggested in its package insert was more efficacious than placebo in the symptomatic treatment of the common cold or flu-like illness. The study drug also reduced the frequency of use of rescue medication as compared with placebo, and was as safe as placebo. These findings suggest that a fixed-dose combination of paracetamol, chlorphenamine and phenylephrine may be an effective and safe alternative for treatment of these clinical entities.

## Abbreviations

ANOVA: Analysis of variance; ECG: Electrocardiogram; FBC: Full blood count; HCPA: Hospital de Clínicas de Porto Alegre; HR: Heart rate; MAO: Monoamine oxidase; NUCLIMED: *Núcleo de Investigação Clínica em Medicamentos*; RCT: Randomized clinical trial; SBP: Systolic blood pressure; V1: Visit 1; V2: Visit 2; V3: Visit 3.

## Competing interests

This study was funded by the manufacturer of the tested product through a research agreement that included reimbursement of all author expenses. The authors declare that the manufacturer did not participate in the design of the study, data collection, statistical planning and analysis, data evaluation and interpretation, or manuscript preparation for publication.

## Authors' contributions

PDP was the principal investigator, and as such was responsible for planning and coordination of the study, statistical analysis and text editing. MCB participated in all phases of the study, from planning to manuscript writing, was responsible for the study logistics and monitoring, and prepared the final version of the paper. RVP prepared the dataset and conducted data qualification, evaluated the final results, conducted statistical analysis, prepared the figures and played a major role in the final editing of the paper. LCCF participated in preparation and implementation of the logistics of the study, including data entry and qualification, CRF review, and database input, and contributed to the final version of the paper. MLS participated in preparation and implementation of the logistics of the study, including data entry and qualification, CRF review, and database input, and contributed to the final version of the paper. ICS was responsible for management and financial planning and execution of the study, participated in preparation and implementation of the logistics of the study, and contributed to the final version of the paper. ADD participated in preparation and implementation of the logistics of the study, including data entry and qualification, CRF review, and database input, and contributed to the final version of the paper. LFCS was the principal co-investigator, responsible for medical review of all patients and data input into CRFs, and contributed to the final version of the paper. All authors read and approved the final manuscript.

## Pre-publication history

The pre-publication history for this paper can be accessed here:

http://www.biomedcentral.com/1471-2334/13/556/prepub
